# A Presentation of Meckel’s Diverticulum With Malignancy

**DOI:** 10.7759/cureus.107758

**Published:** 2026-04-26

**Authors:** Christina A Schott, Hannaka Spillman, Jacob Lambdin, Marian Khalili, Matthew Ng

**Affiliations:** 1 Surgery, George Washington University School of Medicine and Health Sciences, Washington, DC, USA; 2 Colorectal Surgery, George Washington University School of Medicine and Health Sciences, Washington, DC, USA

**Keywords:** abdominal pain, congenital, meckel's diverticulum, surgery, undifferentiated carcinoma

## Abstract

Introduction: Meckel’s diverticulum (MD) is the most common congenital gastrointestinal anomaly resulting from incomplete obliteration of the omphalomesenteric duct. Although often asymptomatic, MD may rarely harbor malignancies. The management of incidentally discovered MD in adults remains controversial.

Case Presentation: A man in his 70s presented with abdominal pain and iron deficiency anemia. Computed tomography demonstrated a 13 × 14 cm heterogeneously enhancing right lower quadrant mass concerning for a primary mesenteric neoplasm. The patient underwent surgical resection, and intraoperative findings revealed a large mass arising from a previously undiagnosed MD. En bloc segmental small bowel resection with associated mesentery was performed. Postoperative recovery was notable for transient ileus. Final pathology demonstrated high-grade undifferentiated carcinoma. Molecular profiling revealed high microsatellite instability (MSI-H) status, programmed death-ligand 1 (PD-L1) positivity, high tumor mutational burden, and an ATM mutation, guiding initiation of immunotherapy.

Discussion: Neoplasms in MD are rare and most commonly neuroendocrine in origin; undifferentiated carcinoma is exceedingly uncommon. This case highlights the diagnostic challenge of MD presenting as a large mesenteric mass and underscores the importance of molecular profiling in identifying actionable biomarkers, particularly in rare malignancies lacking established treatment guidelines. Contemporary evidence supports a selective approach to incidental MD resection, with risk stratification based on factors such as age, sex, and diverticulum size.

Conclusion: Undifferentiated carcinoma should be considered in the differential diagnosis of large mesenteric masses. This case emphasizes the importance of recognizing atypical presentations of MD and the critical role of molecular characterization in guiding personalized therapy when conventional management strategies are limited.

## Introduction

Meckel’s diverticulum (MD) is the most common congenital anomaly of the gastrointestinal tract, arising from incomplete obliteration of the omphalomesenteric (vitelline) duct during the fifth week of embryologic development [1]. It is classically described by the “rule of 2s”-occurring in approximately 2% of the population, measuring ~2 inches in length, and located within ~2 feet of the ileocecal valve-although these features represent variable approximations rather than strict criteria [1,2]. MD may contain ectopic tissue, most commonly gastric or pancreatic, and demonstrates a slight male predominance [1].

While the majority of individuals with MD remain asymptomatic, approximately 4-9% develop complications over their lifetime. Symptomatic presentations most commonly occur in childhood and include gastrointestinal bleeding, obstruction, intussusception, and diverticulitis [1,3]. Neoplasms arising within MD are rare and represent an uncommon cause of symptoms.

Tumors have been reported in approximately 3.2% of MD cases, although this figure includes both benign and malignant lesions. Malignant neoplasms therefore represent a smaller subset of these tumors. Among reported malignancies, neuroendocrine tumors are the most common, followed by gastrointestinal stromal tumors and adenocarcinomas [4]. In contrast, undifferentiated carcinoma arising in MD is exceptionally rare and remains poorly characterized in the literature.

Given the limited data on aggressive non-neuroendocrine malignancies in MD, we present a case of undifferentiated carcinoma to highlight its clinical presentation, diagnostic challenges, and pathologic features. This case was previously presented as an abstract at the 2024 American Society of Colon and Rectal Surgeons (ASCRS) Annual Scientific Meeting.

## Case presentation

A man in his 70s was admitted for evaluation of abdominal pain and iron deficiency anemia. His medical history was significant for stage 3 chronic kidney disease, hypertension, diabetes mellitus, hyperlipidemia, arthritis, and benign prostatic hyperplasia. His surgical history included left knee arthroplasty, trigger finger release, and prior laparoscopic liver resection. He denied tobacco use, reported minimal alcohol intake (1-2 drinks per week), and occasional oral/topical tetrahydrocannabinol use. Family history was notable for uterine cancer in his mother.

On presentation, physical examination revealed a soft, non-tender, and non-distended abdomen without palpable masses. Laboratory evaluation demonstrated iron deficiency anemia with a hemoglobin of 8.4 g/dL, hematocrit of 28.0%, and mean corpuscular volume of 84 fL.

**Figure 1:**
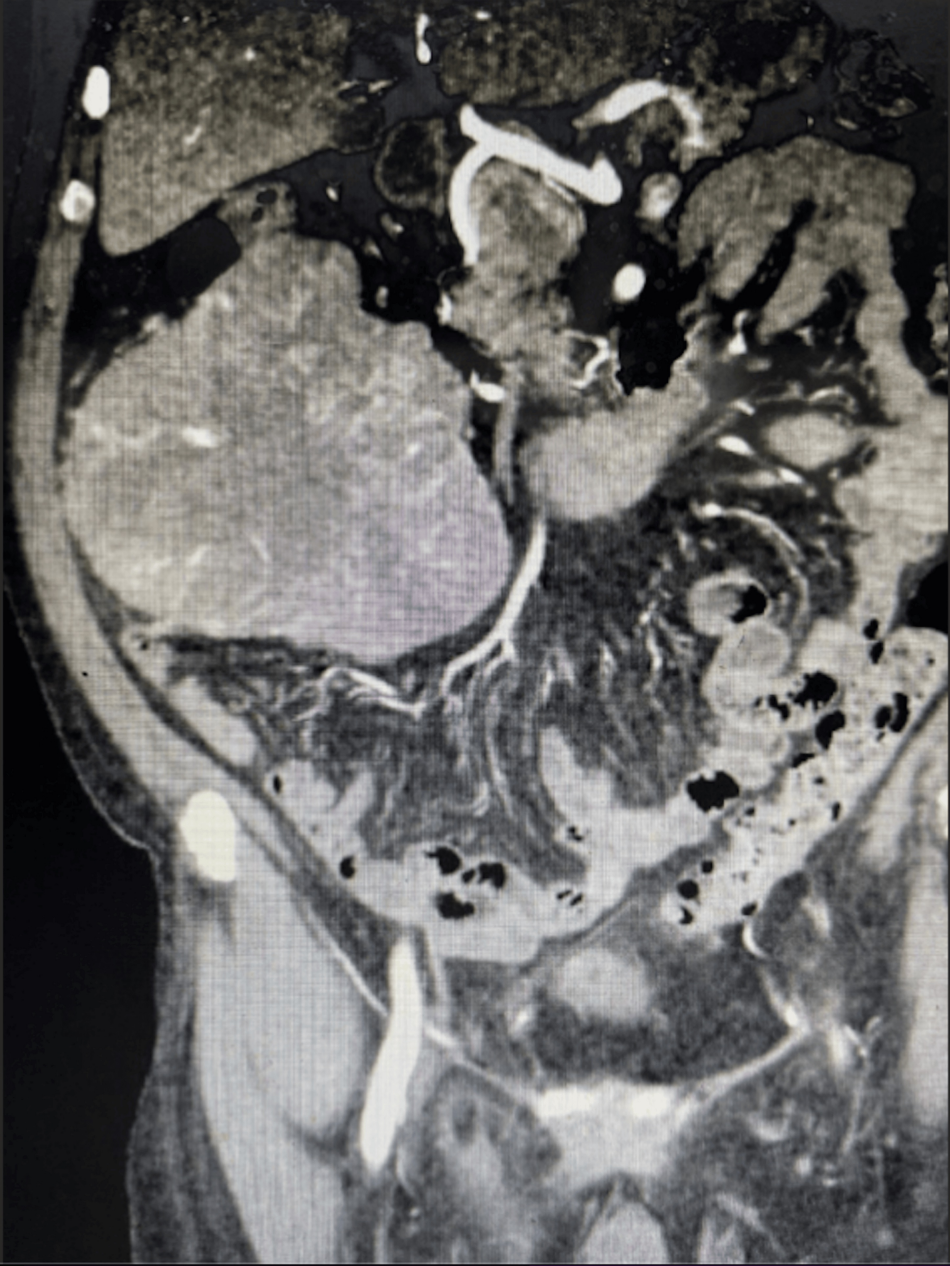
Contrast-enhanced CT scan of the abdomen and pelvis (coronal view) demonstrating a large (13.1 × 9.1 × 14.1 cm) heterogeneously enhancing mass in the right lower quadrant. The lesion exhibits irregular borders, internal heterogeneity, and areas of necrosis, with apparent origin from the mesentery adjacent to the ascending colon. These radiologic features raised concern for a primary mesenteric neoplasm, including sarcoma.

A contrast-enhanced CT scan of the abdomen and pelvis revealed a 13.1 × 9.1 × 14.1 cm heterogeneously enhancing mass in the right lower quadrant adjacent to the ascending colon, suspected to arise from the mesentery (Figure 1). The size, location, and imaging characteristics of the lesion raised concern for a primary mesenteric neoplasm, including sarcoma. Given these findings, further endoscopic evaluation for iron deficiency anemia, including colonoscopy and esophagogastroduodenoscopy, was deferred due to concern for an underlying malignancy requiring surgical management.

Diagnostic reasoning

The differential diagnosis at the time of presentation included mesenteric sarcoma, gastrointestinal stromal tumor, small bowel adenocarcinoma, and neuroendocrine tumor. MD was not strongly suspected preoperatively due to its nonspecific radiographic appearance and the large size of the mass. Preoperative biopsy was deferred given the lesion’s location, the risk of bleeding or tumor seeding, and the likelihood that definitive surgical resection would be required regardless of histopathologic subtype.

**Figure 2:**
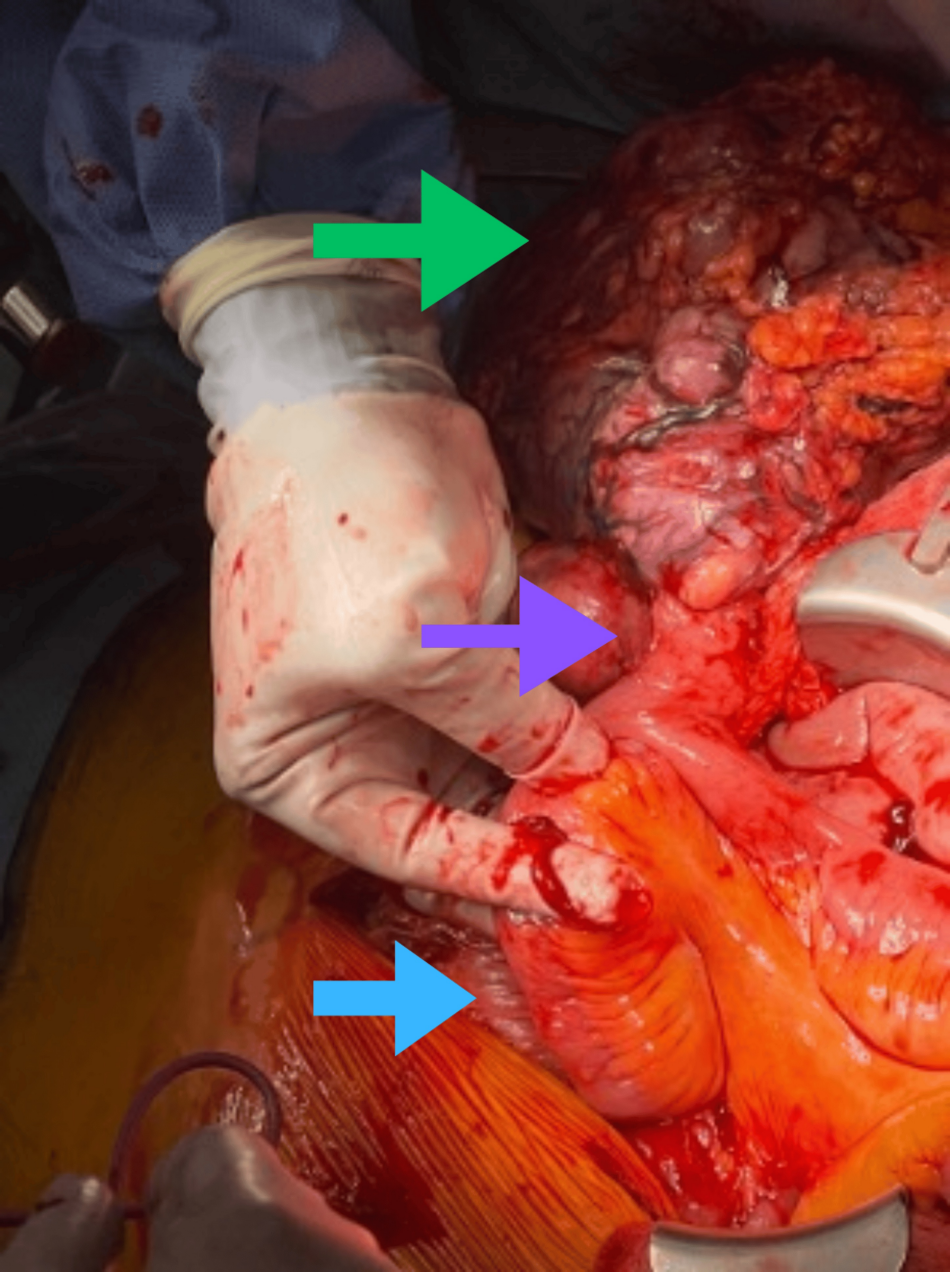
Intraoperative photograph demonstrating a large, hypervascular mass (green arrow) arising from an MD (purple arrow) located on the antimesenteric border of the small intestine. The adjacent small bowel (blue arrow) is shown for orientation. The mass was resected en bloc with segmental small bowel and associated mesentery. MD: Meckel’s diverticulum

Following multidisciplinary discussion, the patient was taken to the operating room for definitive management. Intraoperatively, the mass was found to arise from a previously undiagnosed MD located on the antimesenteric surface of the small bowel (Figure 2). A segmental small bowel resection was performed with en bloc removal of the diverticulum and associated mesentery. The lesion was noted to be hypervascular and irregular in contour but did not involve adjacent structures, including the colon. Adequate proximal and distal margins were obtained, and the mesentery was divided to include associated lymphovascular tissue. The patient tolerated the procedure without intraoperative complications.

On postoperative day 8, the patient developed a prolonged ileus characterized by abdominal distension and intolerance of oral intake, requiring nasogastric tube decompression and initiation of parenteral nutrition. This was likely multifactorial, related to recent bowel resection, perioperative opioid use, and underlying comorbidities including diabetes mellitus and chronic kidney disease. On postoperative day 9, bowel function returned, the nasogastric tube was removed, and the patient was advanced to a clear liquid diet, which he tolerated well. He was subsequently discharged on postoperative day 12.

Final pathology revealed a high-grade undifferentiated carcinoma arising within mesenteric fat associated with an MD. Resection margins were negative for dysplasia or malignancy. Immunohistochemical and molecular analysis demonstrated intact nuclear expression of MSH2, indeterminate MLH1 status, and high microsatellite instability (MSI-H). Programmed death-ligand 1 (PD-L1) expression was positive with a combined positive score (CPS) greater than 10. Additional genomic profiling revealed a p53 mutation, an ATM mutation, and a high tumor mutational burden. Serum CA 19-9 levels were within normal limits.

Given these findings, the patient was referred for oncologic evaluation. Further staging with positron emission tomography (PET) imaging demonstrated an enlarged mildly hypermetabolic right paratracheal lymph node (1.1 × 1.0 cm, standardized uptake value (SUV) 2.5) and a 3 mm non-fluorodeoxyglucose (FDG)-avid pulmonary nodule in the left lower lobe. Based on the tumor’s molecular profile, the patient was initiated on a planned two-year course of immunotherapy and referred for genetic counseling.

Histopathologic slides and PET-CT imaging were not available for inclusion in this report despite attempts to obtain them, representing a limitation in the visual characterization of this case.

## Discussion

We present a rare case of high-grade undifferentiated carcinoma arising in association with a previously undiagnosed MD. Neoplasms in MD are uncommon, with tumors reported in approximately 3.2% of cases; however, this figure includes both benign and malignant lesions. Malignant tumors represent a smaller subset, with some series estimating malignancy rates closer to 5% among MD-associated tumors. Among these, neuroendocrine tumors account for the majority, whereas undifferentiated carcinoma remains exceedingly rare and poorly characterized [5].

Undifferentiated carcinomas of the small intestine are aggressive neoplasms that often present with nonspecific symptoms such as abdominal pain, anemia, gastrointestinal bleeding, and weight loss, contributing to delayed diagnosis [5]. Historically, these tumors have been associated with poor outcomes due to advanced stage at presentation and limited systemic treatment options [6]. However, previously reported survival data largely predate the widespread use of molecular profiling and immunotherapy, limiting their applicability to current clinical practice.

In this case, molecular profiling revealed MSI-H status, PD-L1 expression (CPS >10), high tumor mutational burden, and an ATM mutation. These findings have important therapeutic implications. MSI-H status is a well-established predictive biomarker for response to immune checkpoint inhibitors, reflecting underlying mismatch repair deficiency and increased neoantigen load [7,8]. Similarly, elevated PD-L1 expression may further support the use of PD-1/PD-L1-directed therapies, although its predictive value in small bowel malignancies remains less clearly defined. High tumor mutational burden also correlates with improved responsiveness to immunotherapy across multiple tumor types [9].

The presence of an ATM mutation is additionally notable, as alterations in DNA damage response pathways have been associated with potential sensitivity to poly (ADP-ribose) polymerase (PARP) inhibitors in select malignancies. While the role of PARP inhibition in small bowel and MD-associated carcinomas remains investigational, this finding may have therapeutic relevance in the setting of disease progression or treatment resistance [10,11]. Collectively, these molecular features support the use of immunotherapy in this patient and highlight the growing importance of genomic profiling in guiding treatment decisions for rare gastrointestinal malignancies.

The management of incidentally discovered MD in adults remains controversial [12]. While resection is generally recommended in symptomatic patients, the decision to perform prophylactic diverticulectomy in asymptomatic individuals requires careful consideration. Importantly, prior interpretations of complication risk have been inconsistent. Current literature suggests that incidental diverticulectomy is associated with a relatively low complication rate of approximately 2%, whereas resection performed for symptomatic MD carries a higher risk, reported up to approximately 12%. This distinction is critical when weighing the risks and benefits of prophylactic resection. A review of previously reported cases demonstrates the extreme rarity of undifferentiated carcinoma arising in MD, with only isolated cases described in the literature, most often presenting with nonspecific symptoms and diagnosed at an advanced stage, which can be seen in Table 1 [13-16].

**Table 1:** Reported cases of carcinoma in MD Data derived from [13-16] CEA: Carcinoembryonic antigen; MD: Meckel’s diverticulum

Author	Year	Age/Sex	Presentation	Tumor Type	Treatment	Outcome
Sakoda et al. [15]	2018	91/M	Abdominal pain, perforation, obstruction	Undifferentiated carcinoma	Surgical resection	Not well characterized
Inokuchi et al. [14]	2024	60s/M	Abdominal pain	Adenocarcinoma (ectopic pancreas origin)	Surgery + chemotherapy	Alive at follow-up
Yamaguchi et al. [13]	1989	55/M	Abdominal pain, ↑CEA	Adenocarcinoma	Surgical resection	Not specified
Moore and Parsons [16]	1950	Adult	Abdominal symptoms	Carcinoma (unspecified)	Surgical resection	Not specified
Present case	2024	70s/M	Abdominal pain, anemia, large mesenteric mass	Undifferentiated carcinoma	Surgery + immunotherapy	Ongoing

Although malignancy within MD is rare, it is frequently diagnosed at an advanced stage, contributing to worse outcomes [5]. This risk may be further amplified in the setting of aggressive histologies such as undifferentiated carcinoma. Therefore, selective prophylactic resection in adult patients may be considered on a case-by-case basis, taking into account patient age, comorbidities, intraoperative findings, and overall risk profile.

This case highlights not only the rarity of undifferentiated carcinoma arising in MD but also the evolving role of molecular diagnostics in shaping prognosis and management. As genomic profiling becomes more integrated into oncologic care, previously poor-prognosis tumors may have expanding therapeutic options, underscoring the importance of incorporating molecular data into the evaluation of rare malignancies.

## Conclusions

This case highlights a rare presentation of high-grade undifferentiated carcinoma arising from a previously undiagnosed MD, manifesting as a large mesenteric mass. Such presentations may mimic primary mesenteric neoplasms, and MD should be considered in the differential diagnosis of atypical small bowel or mesenteric masses, particularly when imaging findings are inconclusive.

This case also underscores the critical role of molecular profiling in rare gastrointestinal malignancies. The presence of MSI-H status, PD-L1 expression, and high tumor mutational burden supported the use of immunotherapy, while the identification of an ATM mutation may have future therapeutic implications. In the absence of established treatment guidelines for undifferentiated carcinomas of Meckel’s origin, these biomarkers provide important guidance for personalized oncologic management.

Finally, the management of incidentally discovered MD in adults should be individualized. Prophylactic resection may be considered in patients with risk factors for complications or malignancy, including male sex, age younger than 50 years, and diverticulum length greater than 2 cm.

Overall, this case emphasizes that undifferentiated carcinoma, although exceedingly rare, should be considered in the evaluation of large mesenteric masses, and that comprehensive molecular characterization is essential to guide management when conventional treatment pathways are limited.
